# Parotid Acinic Cell Carcinoma as a Presentation of Birt-Hogg-Dube Syndrome

**DOI:** 10.7759/cureus.36074

**Published:** 2023-03-13

**Authors:** Jason Xin, Aaron Goffinet, Steven Machusko, Ramy Shoela

**Affiliations:** 1 Radiology, Saint Louis University Hospital, Saint Louis, USA

**Keywords:** oncocytoma, parotid tumor, birt-hogg-dube, bhd, acinic cell carcinoma

## Abstract

Birt-Hogg-Dube syndrome (BHD) is a rare autosomal dominant disease classically associated with fibrofolliculomas, pulmonary cysts, spontaneous pneumothorax, and renal cancers. Information about its manifestation aside from the ones listed prior is limited. There have been several reports of BHD associated with parotid oncocytomas and rare benign epithelial tumors. Here, we report the first known case of BHD in association with parotid acinic cell carcinoma, a rare low-grade malignant tumor of salivary glands.

## Introduction

Birt-Hogg-Dube syndrome (BHD) is a rare autosomal dominant disease classically associated with skin fibrofolliculomas, pulmonary cysts, spontaneous pneumothorax, and renal cancers [1]. It is associated with a mutation in the Folliculin (FLCN) gene, a tumor suppressor gene that encodes for the protein folliculin, shown to be expressed in the skin, distal nephron of the kidney, stromal cell and pneumocytes of the lung, and acinar cells of the pancreas and parotid gland [2]. There have been several case reports of BHD associated with parotid tumors, most commonly parotid oncocytoma. Eight cases of parotid oncocytoma have been reported [3]. Here, we present the first case of parotid acinic cell carcinoma in a patient with proven BHD through genetic testing, highlighting a possible association between BHD and parotid acinic cell carcinoma. 

## Case presentation

**Figure 1:**
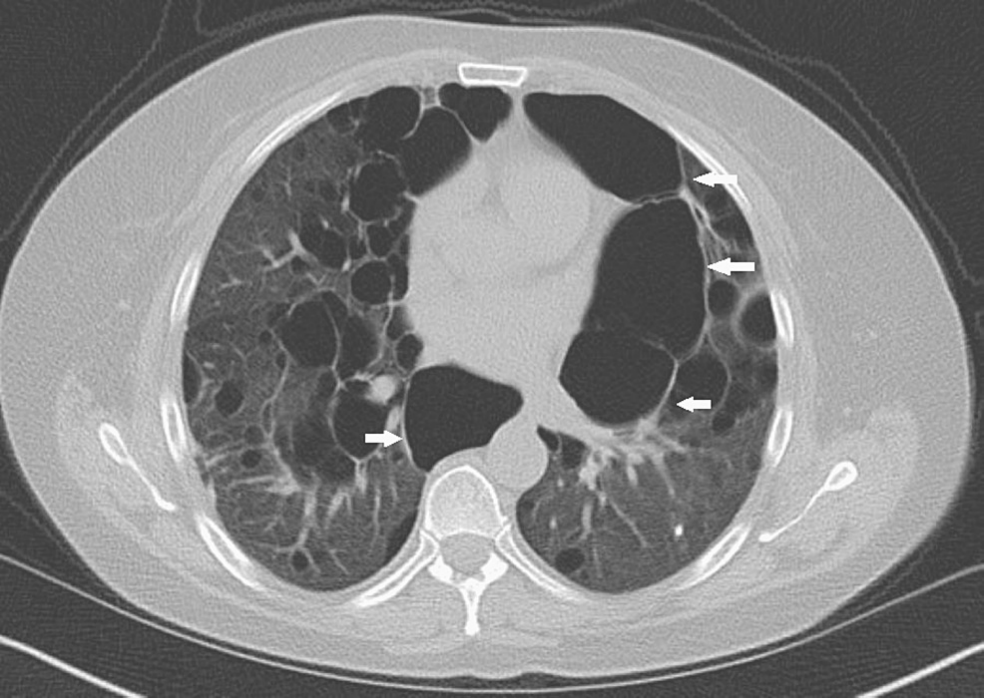
CT chest without contrast showing multiple large bullae without focal consolidation, pleural effusion, or focal pleural thickening.

A 58-year-old woman initially presented for evaluation of a right leg mass and a right superficial lobe parotid lesion in December 2018. The initial fine needle aspiration of the parotid mass was suggestive of oncocytoma. The right leg mass was biopsied and showed high-grade pleomorphic sarcoma which was subsequently resected, and the patient received adjuvant radiotherapy. A chest CT was obtained to evaluate for metastasis, which showed multiple large biapical, paraseptal, and bibasilar bullae (Figure 1). The patient had no history of smoking or IV drug use and no prior family history of pulmonary disease. 

In 2020, the patient reported continued pain secondary to the parotid mass, and a parotidectomy was subsequently performed. Microscopic examination showed a solid neoplasm with a thin capsule in most areas with cells arranged in a sheeted pattern with packets of tumor cells interspersed with delicate vessels. Cytologically, the neoplastic cells had an abundant pale granular to eosinophilic cytoplasm with eccentric ovoid vesicular nuclei. The tumor morphology was similar to that seen in the patient’s prior fine needle aspiration, in which at that time, the tumor cells were negative for thyroid transcription factor 1 (TTF1), renal cell cancer (RCC) marker, and vimentin, and pan-cytokeratin positive. Immunostains from the current tumor cells showed tumor cells positive for epithelial cell adhesion molecule BerEp4 and negative for GATA binding protein 3 (GATA-3), tumor protein 63 (p63), and Paired box gene 8 (PAX8), which essentially ruled out salivary gland oncocytoma and metastatic renal cell carcinoma. The pathologic diagnosis was acinic cell carcinoma.

The patient continued to be worked up for cystic lung disease. Lymphangioleiomyomatosis was ruled out given normal vascular endothelial growth factor (VEGF), alpha-1-antitrypsin deficiency was ruled out by normal AA genotype, and the autoimmune workup was negative. The patient was then suspected and tested for BHD via genetic testing. Informed consent was obtained from the patient for FLCN genetic testing. A heterozygous c.117705_1177-3del intronic deletion in the FLCN gene was identified, confirming the diagnosis of BHD. 

## Discussion

BHD is a rare disease, and there is limited information regarding the manifestations of BHD apart from cutaneous manifestations, pulmonary cysts, and renal tumors. It is caused by a germline mutation in the FLCN gene located on chromosome 17p11.2, and is expressed in the skin, distal nephron of the kidney, stromal cells and pneumocytes of the lung, and acinar cells of the pancreas and parotid gland [2]. 

There have been several reports of BHD associated with parotid tumors, most notably parotid oncocytoma, a rare benign epithelial tumor. Eight cases of parotid oncocytoma have been reported [3]. In addition to parotid oncocytoma, Palmirotta et al reported on 2 BHD families and identified one woman who had a parotid pleomorphic adenoma at age 43 [4]. In 2011, Maffe et al reported on 19 patients with suspected BHD and identified a Warthin parotid tumor occurring in a 59-year-old male [5]. To our knowledge, our patient is the first case of BHD in association with parotid acinic cell carcinoma. 

Acinic cell carcinoma is a low-grade malignant salivary neoplasm that constitutes 17% of primary salivary gland malignancies and approximately 6-8% of all salivary gland neoplasms, most commonly occurring in the parotid gland. The median age of diagnosis is 52 years, younger than that for most other salivary gland cancers [6]. The genetic alterations linked to acinic cell carcinoma of the parotid gland include alterations at chromosomes 4p, 5q, 6p, and 17p, which is also where the FLCN gene is located [7].

The identification of acinic cell carcinoma in BHD syndrome is significant given its tendency to recur and produce metastases, and may have an aggressive evolution [8]. The average recurrence rate amongst several studies was estimated to be around 35%, and late local recurrence was also reported in many cases up to 30 years from the initial presentation [9]. Due to the notably high tendency of acinic cell carcinoma to recur and produce latent metastasis, long-term follow-up is mandatory after treatment, which generally consists of total or subtotal parotidectomy. There is no survival advantage with adjuvant radiotherapy in low-grade and early-stage tumors if resection is complete, but the benefit in higher-grade or higher-stage tumors is unclear. The benefit of adjuvant radiotherapy in cases of positive surgical margins is also uncertain [10]. In addition, acinic cell carcinoma has been considered chemotherapy-resistant, most likely due to the usually slow metabolism of this cancer.

## Conclusions

Birt-Hogg-Dube syndrome is a rare disorder, with manifestations generally involving the skin, lungs, and kidneys. While a few cases of parotid oncocytoma have been reported in association with BHD, no previous reports of parotid acinic cell carcinoma have been reported. Given the expression of FLCN mRNA in acinar cells of the pancreas, there is a possible association between BHD syndrome and acinic cell carcinoma. Due to its increased tendency to recur, long-term monitoring is very important after excision. Further investigation of the impact and benefits of adjuvant radiotherapy in cases of higher-grade or higher-stage tumors is merited. 
